# Complementarity of End Regions Increases the Lifetime of Small RNAs in Mammalian Cells

**DOI:** 10.1371/journal.pone.0044157

**Published:** 2012-09-12

**Authors:** Anastasia P. Koval, Irina K. Gogolevskaya, Karina A. Tatosyan, Dmitri A. Kramerov

**Affiliations:** Engelhardt Institute of Molecular Biology, Russian Academy of Sciences, Moscow, Russia; German Cancer Research Center, Germany

## Abstract

Two RNAs (4.5SH and 4.5SI) with unknown functions share a number of features: short length (about 100 nt), transcription by RNA polymerase III, predominately nuclear localization, the presence in various tissues, and relatively narrow taxonomic distribution (4 and 3 rodent families, respectively). It was reported that 4.5SH RNA turns over rapidly, whereas 4.5SI RNA is stable in the cell, but their lifetimes remained unknown. We showed that 4.5SH is indeed short-lived (t_1/2_∼18 min) and 4.5SI is long-lived (t_1/2_∼22 h) in Krebs ascites carcinoma cells. The RNA structures specifying rapid or slow decay of different small cellular RNAs remain unstudied. We searched for RNA structural features that determine the short lifetime of 4.5SH in comparison with the long lifetime of 4.5SI RNA. The sequences of genes of 4.5SH and 4.5SI RNAs were altered and human cells (HeLa) were transfected with these genes. The decay rate of the original and altered RNAs was measured. The complementarity of 16-nt end regions of 4.5SI RNA proved to contribute to its stability in cells, whereas the lack of such complementarity in 4.5SH RNA caused its rapid decay. Possible mechanisms of the phenomenon are discussed.

## Introduction

Most types of RNA in mammalian cells are stable: their lifetime is estimated at hours or even days. However, some mRNAs are short-lived and their half-life (t_1/2_) is estimated at minutes. Short lifetime of such mRNAs is due to the need to quickly change the cellular level of proteins encoded by them (e.g., factors regulating cell cycle and proliferation). The rapid degradation is usually due to the presence of AU-rich elements in mRNA [1]. Mechanism and regulation of such mRNA degradation have been studied rather well [2], [3]. At the same time, the degradation of small RNAs, except for tRNA [4], [5] and microRNA [6], remains unexplored.

Mammalian small RNAs (70–300 nt) were discovered in the 70 s of the last century [7]–[9]. It gradually became clear that they play an important role in pre-mRNA splicing (U1, U2, U4, U5, U6, U11, and U12 RNAs), pre-rRNA processing (U3 and U14 RNAs) and modification (C/D box RNAs and H/ACA box RNAs), as well as protein secretion (7SL RNA), transcription regulation (7SK RNA), and the initiation of DNA replication (Y RNAs) [10]–[14]. In contrast to these RNAs that are apparently ubiquitous for all eukaryotes, there are small RNAs with a relatively narrow range of distribution (stenoRNAs) [15].

The most studied among them is BC1 RNA that is synthesized by RNA polymerase III (pol III) in the nervous tissue and testes of (presumably) all rodents, but not other mammals [16], [17]. Usually, the genome has a single BC1 gene, which is a master gene of ID [18], a repetitive DNA sequence belonging to short interspersed elements (SINEs) [19]. BC1 RNA was shown to repress translation of certain mRNAs in dendrites [20]. BC200 RNA has a similar function in humans and, likely, other higher primates [20], although it originated from FLAM-C, the ancient monomeric Alu SINE [21].

The two RNAs studied here, also belong to the stenoRNA group. 4.5SI RNA is present in rodents of only three related families: Muridae (mice, rats, gerbils), Cricetidae (hamsters, voles), and Spalacidae (mole rats, root rats, zokors) [15], [22]. Tissues of the same rodents, as well as jerboas and birch mice (Dipodidae), contain 4.5SH RNA [23]. These RNAs have similar length (4.5SI, 98–101 nt and 4.5SH, 94 nt) [15], [24], [25] and are localized predominately in the nucleus. In mouse, 4.5SI is transcribed from three genes located on chromosome 6 and spaced 40 kb apart [26]. The number of 4.5SH genes is much higher (700–800) in genomes of all rodents studied; each 4.5SH gene is a part of a 4–5 kb tandemly repeated unit [23], [27]. The both of RNAs are evolutionarily related to SINEs: 4.5SI RNA genes seemed to originate from SINE B2 [28], whereas 4.5SH RNA genes were derived from a copy of an ancient SINE pB1 [29]. Genes of these RNAs, as well as SINEs, are transcribed by pol III due to an internal promoter composed of two parts (boxes A and B), while 5′-flanking sequences also influence transcription of these genes [26], [30]. The function of both RNAs remains unknown, although an association of 4.5SH RNA with poly(A)-containing RNAs was shown [27], [31]. Contrary to most of mammalian small RNAs, 4.5SH RNA has a rapid turnover in mouse cells [27]. Here we constructed chimeric 4.5SH/4.5SI RNAs, as well as modified sequences of the two RNAs by other ways, and showed that the complementarity of end regions of 4.5SI RNA contributes to its stability in cells, whereas the lack of such complementarity in 4.5SH RNA causes its rapid decay.

## Results

### Study of 4.5SH and 4.5SI RNA stability

**Figure 1:**
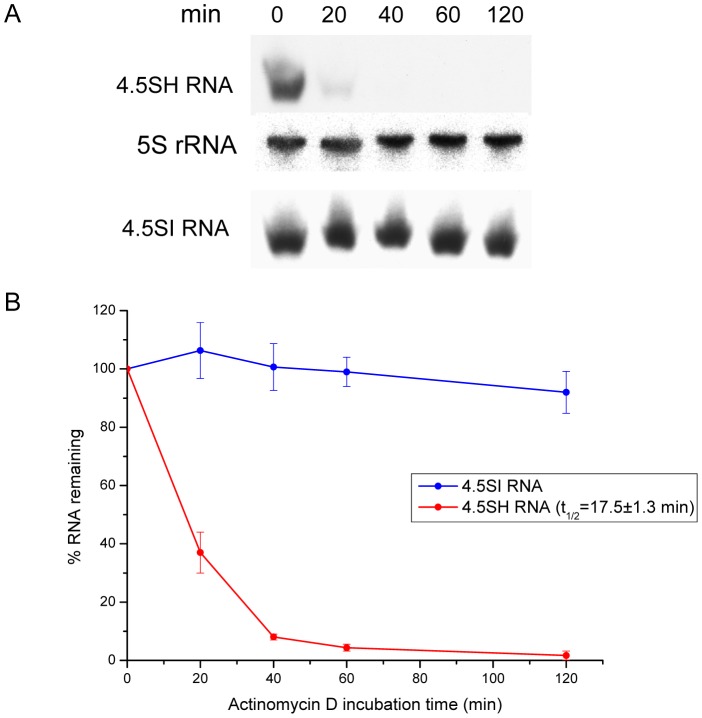
Determination of 4.5SH RNA half-life. (**A**) Detection of 4.5SH and 4.5SI RNA by Northern hybridization in total RNA isolated after the addition of actinomycin D to KAC cells. 5S rRNA was used as a loading control. (**B**) Graphs showing the rapid decay of 4.5SH RNA and the stability of 4.5SI RNA. Each graph is based on data from three experiments (error bars, s.d.).

**Figure 2:**
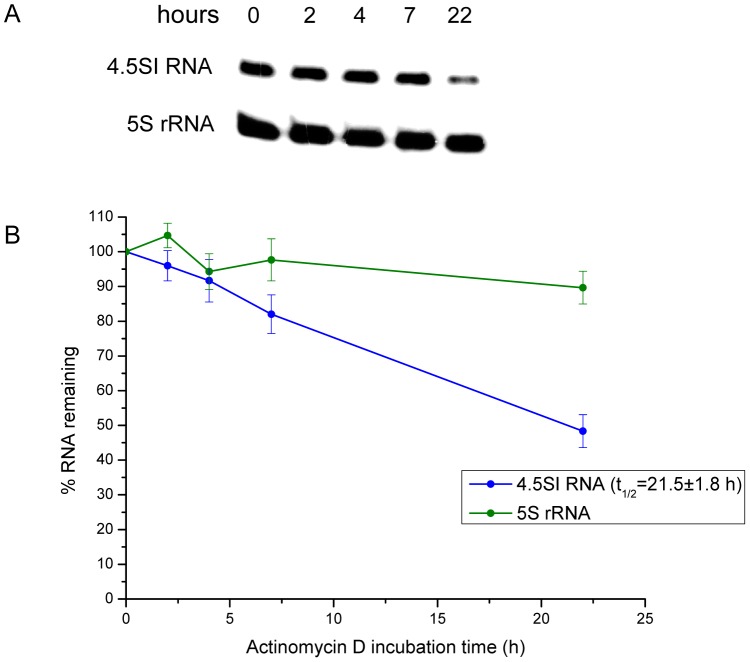
Determination of 4.5SI RNA half-life. (**A**) Northern hybridization of 4.5SI RNA and 5S rRNA in total RNA isolated from KAC cells exposed to actinomycin D for different time periods. (**B**) Graphs demonstrating the slow decrease of the 4.5SI RNA level in cells following the start of transcription inhibition. Highly stable 5S rRNA was used as a loading control. Error bars, s.d., N = 3.

Schoeniger and Jelinek [27] studied the kinetics of [^3^H]uridine incorporation in 4.5SH RNA and concluded that this RNA has a rapid turnover in murine erythroleukemia cells. However, the lifetime of 4.5SH RNA was not estimated in those experiments. We have not found reports on 4.5SI RNA lifetime, although Ro-Choi et al. [24] suggested its stability in cells. We decided to test the established view on the turnover rate of the two RNAs and used the simple and reliable method based on transcription inhibition by actinomycin D. Figures 1 and 2 show the results of the detection of 4.5SH and 4.5SI RNA isolated from murine Krebs ascites carcinoma (KAC) cells at different time points following the addition of actinomycin D to cell culture media. The data demonstrate instability of 4.5SH RNA and stability of 4.5SI RNA and allow us to estimate their half-life as 18 min and 22 h, respectively.

### Study of structural features determining the lifetime of 4.5SH and 4.5SI RNA

**Figure 3:**
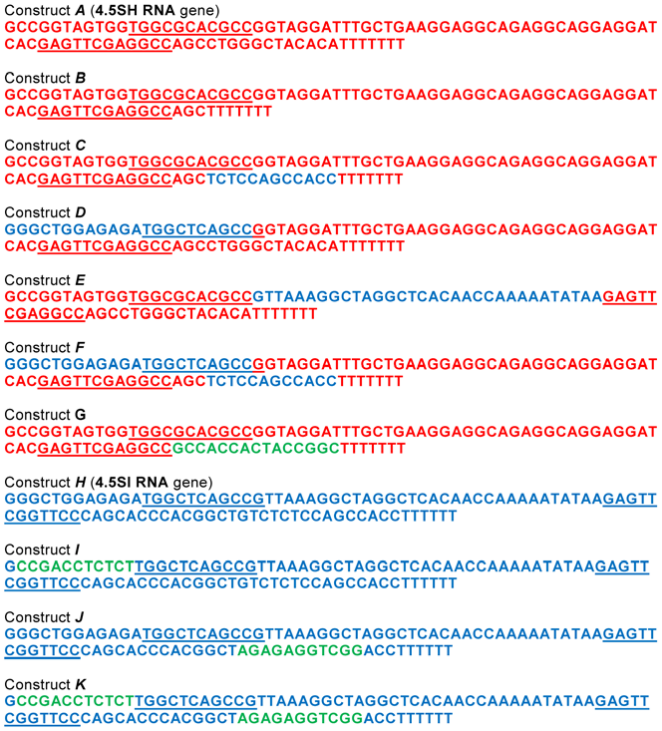
Nucleotide sequences of 4.5SH and 4.5SI RNA genes as well as their derivatives. Nucleotides of 4.5SH and 4.5SI RNA sequences are shown in red and blue, respectively. Green letters correspond to altered nucleotide sequences. Boxes A and B of the pol III promoter are underlined. T_6–7_ at the end of constructs are the pol III terminator.

**Figure 4:**
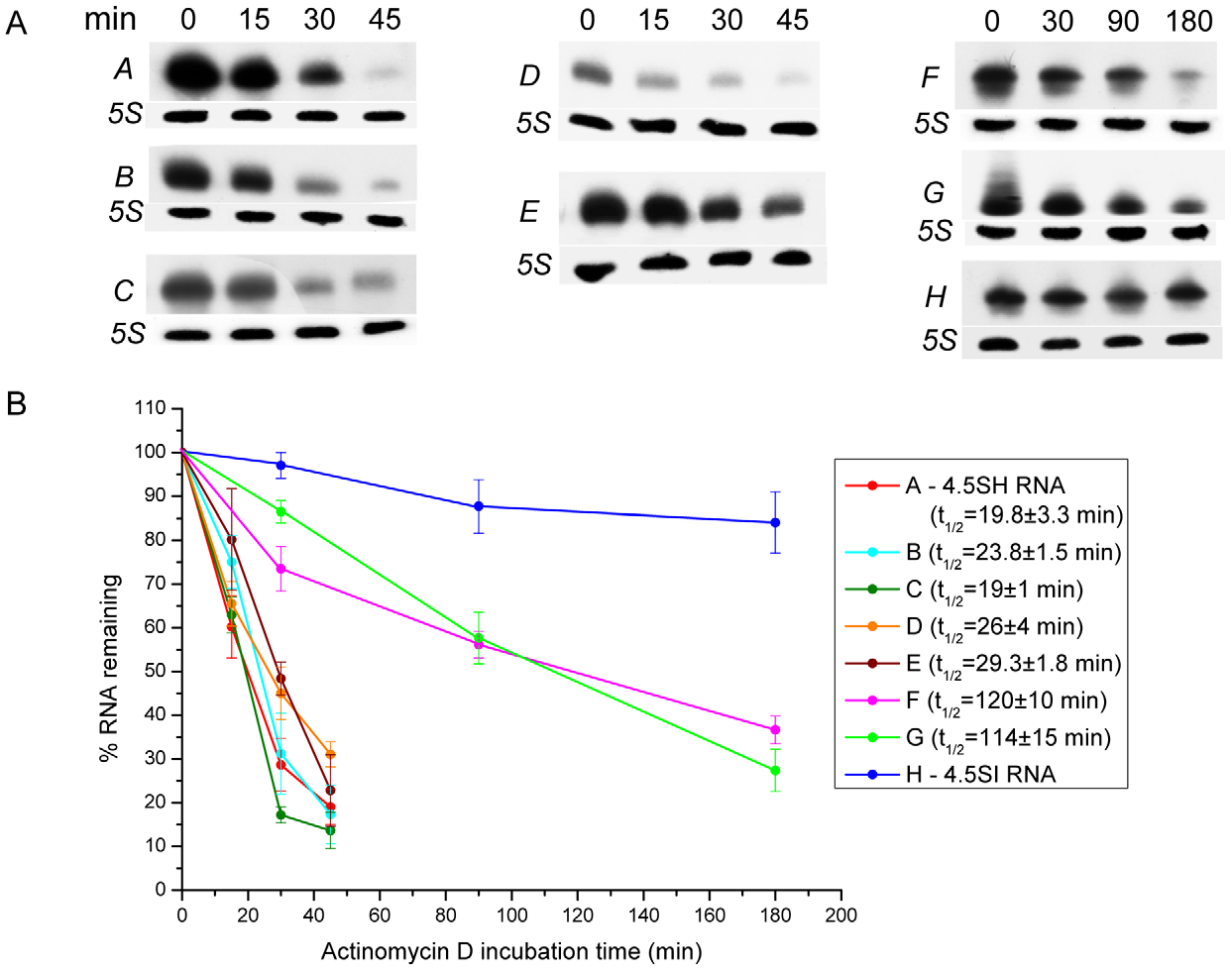
Determination of half-life of 4.5SH RNA and its derivatives in transfected HeLa cells. (**A**) Detection of 4.5SH RNA, its derivatives, 4.5SI RNA and 5S rRNA (loading control) by Northern hybridization. (**B**) Decay kinetics of RNAs transcribed from transfected constructs. Each graph is based on data from three transfection experiments (error bars, s.d.). Average half-lives with standard deviations for RNAs transcribed from each construct except *H* (long-living 4.5SI RNA) are presented.

One can suggest that peculiarities of structure determine the difference in stability between 4.5SH and 4.5SI RNAs in the cell. To examine this hypothesis, we created plasmid constructs containing chimeric genes of 4.5SH and 4.5SI RNAs (Fig. 3). The transcription of such genes resulted in the formation of 4.5SH RNA molecules in which some parts were replaced by the corresponding regions of 4.5SI RNA. HeLa cells (human carcinoma) were transfected with the plasmid constructs obtained, and in 20 h, the half-life of the synthesized RNA was evaluated using the treatment of cells with actinomycin D. Natural and chimeric 4.5SH/4.5SI RNAs were detected by Northern hybridization in total RNA preparations isolated in different periods of time following administration of actinomycin D (Fig.4A, B). The results show that the deletion of 3′end region (construct *B*), as well as the replacement of 3′ end (construct *C*), 5′ end (construct *D*) or central (construct *E*) regions of 4.5SH RNA with corresponding regions of 4.5SI RNA did not significantly change the stability of these RNAs, in comparison with natural 4.5SH RNA (construct *A*). However, the simultaneous replacement of 3′ and 5′ end regions in 4.5SH RNA (construct *F*) by the corresponding regions of 4.5SI RNA essentially increased the lifetime of the chimeric RNA. Its half-life was estimated as 2 h (Fig. 4C).

**Figure 5:**
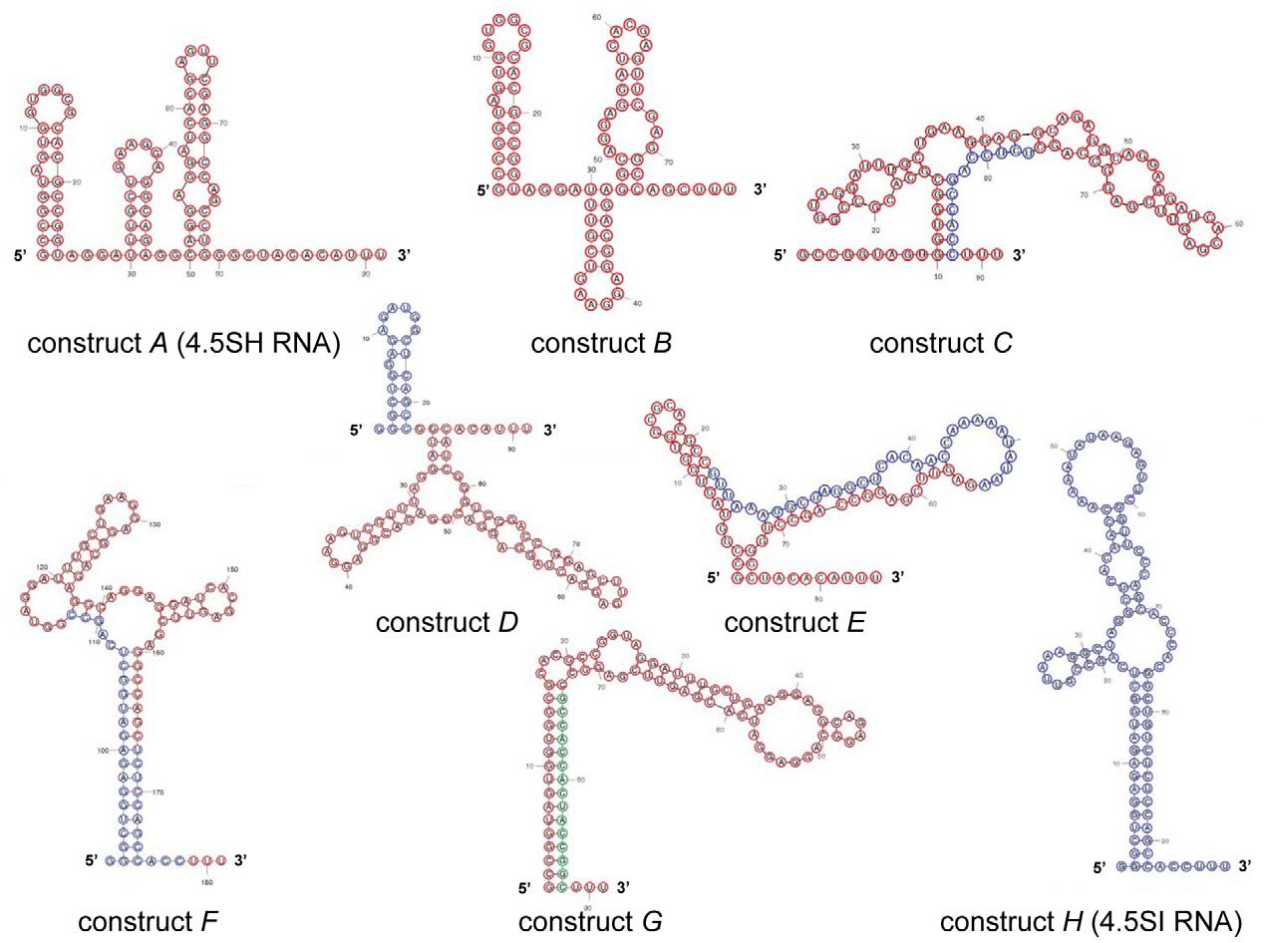
Predicted secondary structures of 4.5SH RNA (*A*) and its derivatives (*B–G*) as well as 4.5SI RNA (*H*). 4.5SH- and 4.5SI-derived nucleotides are marked by red and blue circles. Nucleotides of altered sequences are marked by green circles.

These data allowed us to put forward two hypotheses. According to the first one, the stability of 4.5SI RNA is determined by its interaction with proteins recognizing the specific nucleotide sequences at 5′ and 3′ ends of this RNA. The second hypothesis is based on the prediction of secondary structures of the natural and chimeric 4.5SH/4.5SI RNAs (Fig. 5). We suggested that 4.5SI RNA stability is due to the complementary interaction between 5′ and 3′ end regions of this RNA, which results in formation of long (16 pb) double-helical stem. Such complementary interaction between the termini is absent in 4.5SH RNA and all chimeric RNAs, except for RNA transcribed from the construct *F* (Fig. 5). To choose between these two hypotheses, we made an additional construct that contained 4.5SH RNA gene with 13 nucleotide substitutions at the 3′ end region; due to these substitutions the 3′ region became complementary to the 5′ region (Figs. 3 and 5, construct *G*). The half-life of the RNA obtained was estimated as 2 h (Fig. 4B). This result conforms with the second hypothesis, i.e. it is the complementarity between RNA end regions that increases its lifetime in the cell.

**Figure 6:**
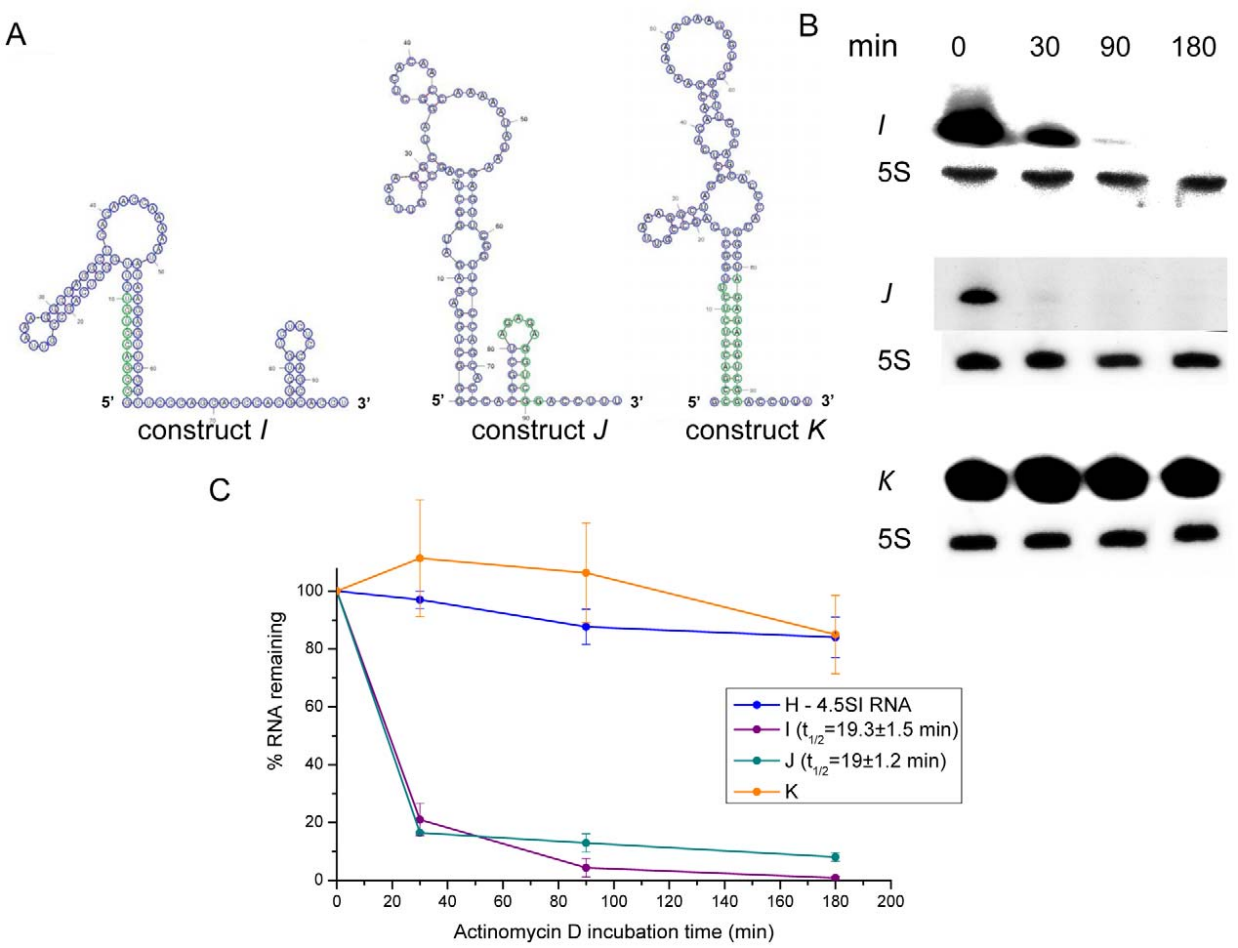
The impact of the end stem on the 4.5SI RNA lifetime. (**A**) Predicted secondary structures of 4.5SI-derived RNAs. (See the nucleotide sequences of the corresponding constructs in Fig. 3). Designations are the same as in Fig. 5. (**B**) Detection of RNA transcribed from the constructs *I*, *J*, and *K* in HeLa cells treated with actinomycin D. 5S rRNA was used as a loading control. (**C**) Decay kinetics of RNA transcribed from constructs *I*, *J*, *K*, and *H* (original 4.5SI RNA). Each graph is based on data from three transfection experiments (error bars, s.d.). Average half-lives with standard deviations only for short-living RNAs (*I* and *J*) are presented.

4.5SI RNA was studied in a similar way. In this RNA, 11-nt regions at 5′ and 3′ ends were changed separately or together (Fig. 3). The sequence replacement at one of the ends (constructs *I* and *J*) resulted in disruption of the end complementarity (Fig. 6A) and dramatically decreased the RNA lifetime (t_1/2_∼20 min) (Fig. 6B,C). On the contrary, the sequence replacement of both ends (construct *K*) recovered the complementarity and rendered RNA stable (Fig. 6A,B).

Thus, the secondary structure (rather than the nucleotide sequence of 5′ and 3′ ends) is crucial for stability of 4.5SI RNA in the cell. Short lifetime of 4.5SH RNA is a result of the lack of the complementarity between its end regions, whereas other mechanisms seem not to be involved in stabilizing this RNA in the cell.

## Discussion

4.5SH RNA belongs to the rare type of small RNAs that is characterized by short lifetime (t_1/2_∼18 min in KAC cells). In human, chimpanzee, and gorilla (but not in other primates), a 117 nt RNA, SnaR (small NF90-associated RNA), was found [32]. Half-life of the RNA is 15 min [33]. SnaR genes derived from Alu-related SINEs seem to have originated from 7SL RNA. These features of SnaR resemble those of 4.5SH RNA (its genes are the derivatives of SINE pB1, and also originated from 7SL RNA). Thus, primates SnaR and rodent 4.5SH appeared as a result of parallel evolution. However, in contrast to 4.5SH RNA, SnaR is expressed at high levels only in the testis, whereas in other organs its content is very low or moderate (brain). A fraction of SnaR is associated with ribosomes or polysomes, which can point to the involvement of this RNA in the translation control [33]. We, however, did not observe the association of 4.5SH RNA with polysomes (K. Tatosyan and D. Kramerov, unpublished data).

Pol III transcripts of SINEs (that are unable for polyadenylation) are also short-lived small RNAs. Pol III transcripts of murine SINE B2 without a polyadenylation signal (AAUAAA) are rapidly degraded in the cell (t_1/2_∼40 min), whereas polyadenylated B2 transcripts are stable [34]. Most likely, poly(A)-binding protein (PABP) protects poly(A)-containing SINE RNA from degradation by 3′endonucleases.

One can expect that other short-lived small RNAs will be found. Difficulty in their discovery can be related to low copy numbers of such RNAs in the cell. Despite the short lifetime of 4.5SH RNA, the number of its molecules in the cell is rather large (13,000), apparently due to the large number (700–800) of genes encoding this RNA in the mouse genome [27]. However, the number of genes of other short-lived small RNAs can be much smaller, which can result in very small number of molecules of the small RNAs in the cell (see as example mouse MEN β tRNA-like small RNA [5]). Interestingly, by depleting RNA degradation machinery, Preker and coworkers found so called promoter upstream transcripts (PROMPTs) that are various, low-copy, non-coding, and very rapidly decaying RNAs [35].

Here we searched for RNA structural features which determine the short lifetime of 4.5SH, as compared with the lifetime of 4.5SI RNA (t_1/2_∼22 h). These RNAs demonstrate a number of similar traits: length, synthesis by pol III, predominately nuclear localization, the presence in various tissues, relatively narrow taxonomic distribution, and the evolutionary relationship with SINEs. *A priori* the great difference between lifetimes can be due to the difference of nucleotide sequences of these two RNAs. For example, 4.5SH RNA could contain degradation signals, much as short-lived mRNAs carry AU-rich elements. On the other hand, 4.5SI RNA could contain nucleotide sequences that can specifically bind proteins preventing rapid degradation of the RNA.

We found that replacing the nucleotide sequences of 4.5SH RNA with the corresponding regions of 4.5SI RNA did not significantly increase the lifetime of the chimeric 4.5SH RNA. These results argue against the hypothesis that the primary structure of some regions determines the lifetime of these two RNAs. On the other hand, the simultaneous replacement of 22 nt at 5′ end and 12 nt at 3′ end (construct F) increased the RNA lifetime by 6-fold. According to the secondary structure prediction, this double replacement results in the formation of a double-helical stem (16 bp with a single mismatch) that includes both termini of the RNA. A similar double-helical stem (but without the mismatch) can be predicted for 4.5SI RNA (Fig. 5*H*). We suggested that such stem formed by RNA termini increases lifetime of RNA. To test it, the 15-nt region at 3′ end of 4.5SH RNA was replaced by the sequence complementary to 5′ end of this RNA (construct *G*). It was found that the lifetime of this RNA is also 6-fold longer than that of the original 4.5SH RNA. This result proves that it is the complementarity of the ends of the RNA, rather than primary structure per se, that increases the lifetime of the RNA.

A question may arise: why in these experiments, the lifetime of RNA is increased by 6 times (t_1/2_∼2 h), and not many times more? Perhaps, other elements of secondary structure (in addition to 16-bp stem) also contribute to the long lifetime of 4.5SI RNA. In other words, probably only the entire secondary structure of genuine 4.5SI RNA ensures its long lifetime (t_1/2_∼22 h). One can speculate that some proteins recognize the RNA shape formed by its secondary structure and prevent its decay. However, this complex issue remains unexplored. At the same time, by changing 11-nt end regions to mutually complementary sequences, we clearly showed the importance of complementarity of ends in determination of 4.5SI RNA lifetime. The change of just one end (constructs *I* and *J*) resulted in drastic decrease of the lifetime of the modified 4.5SI RNA (t_1/2_∼20 min), whereas the replacement of both ends preserved the RNA stability at the level similar to that of native 4.5SI RNA.

Here we have not studied the secondary structure of RNA by enzymatic methods. There are no reports on the enzymatic study of 4.5SI RNA folding. We believe that this lack of knowledge is not very significant in the context of this study: computer prediction of the secondary structure of this RNA is quite reliable as it contains a long (16 bp) perfect stem (Fig. 5, construct *H*). Labuda and Zietkiewicz [36] studied the secondary structure of 4.5SH RNA using enzymatic methods, however, they encountered some difficulties. On one hand, these authors revealed the same two hairpins that are present in the predicted structure of 5′ half of this RNA (Fig. 5A). On the other hand, contradictory results were obtained for 3′ half of 4.5SH RNA: the same sites were often cut by both single-strand- and double-strand-specific nucleases. It was interpreted as the coexistence of many conformational forms [36]. Thus, 4.5SH RNA does not have long perfect hairpins and its secondary structure is very dynamic.

The way of RNA stabilization described here is not ubiquitous, as many small RNAs have no double-helical stem formed by complementary interaction between end regions. For example, BC1 RNA is quite stable but does not demonstrate the complementarity between two end regions; on the contrary, this RNA has long and short stem-loop structures at 5′ and 3′ ends, respectively [37]. Additionally, BC1 RNA contains a long A-rich region located immediately upstream of 3′-stem; PABP can bind this region [38] and contribute to stability of BC1 RNA in cells. On the other hand, 7–12 bp stems formed by complementary interaction between 5′ and 3′ end regions are present in tRNAs, 5S rRNA, Y1-5 RNAs etc. A single nucleotide mismatch in the acceptor stem dramatically reduces the level of the tRNA in mouse cells [5]. Thus, the 7 bp-acceptor (end) stem is a possible factor of tRNA stability in cells. It was found that two CCA trinucleotides were synthesized at the 3′ end of tRNA with the single nucleotide mismatch, and such modification resulted in a rapid decay of the RNA [5]. It seems unlikely that the same mechanism is involved in the rapid decay of 4.5SH RNA because: (i) posttranscriptional CCA addition is specific for tRNA and tRNA-like transcripts; (ii) cDNA sequencing revealed no CCA trinucleotides at 3′-end of 4.5SH RNA [23].

The mechanism of stabilization of small RNAs by formation of end stem remains unclear. One can suggest that such a double-helical structure protects RNA from exonucleases (e.g. Xrn1) and exosomes [39], [40]. Perhaps, proteins binding to long double-helical stems are also involved in the prolongation of small RNA lifetime. There are a number of proteins with various functions that can specifically bind to double-stranded RNA [41], [42]. These issues can be studied using the system of short- and long-lived RNAs that was described by us.

## Materials and Methods

### Cells cultivation, transfection, inhibition of transcription, RNA isolation

Krebs ascites carcinoma II cells [43] were suspended in Dulbecco's modified Eagle's medium (DMEM) containing 10% fetal calf serum and 10 mM Hepes, pH 7.0. Cells (10^7^ per ml) were gently stirred at 37°C in a vial with a tightly closed cap. Inhibitor of transcription, actinomycin D (5 µg/ml), was added to medium and 5 ml aliquots were taken in different time periods. Cells were collected by centrifugation and total cellular RNA was isolated using the guanidinium thiocyanate method [44].

Hela cells were grown to 80% confluency in 60 mm Petri dishes using DMEM with 10% fetal calf serum. Cells were transiently transfected with 4.5 µg of plasmid DNA applying TurboFect *in vitro* Transfection Reagent (Fermentas, Vilnius, Lithuania) following the manufacturer's protocol. Actinomycin D (5 µg/ml) was added to cells 20 h post-transfection. Then total cellular RNA was isolated in different time periods (0 to 180 min). Following ethanol precipitation, RNA was treated with 100 µg/ml RNase-free DNase I at 37°C for 30 min and purified by chloroform extraction. Ethanol-precipitated RNA was dissolved in 40 µl 0.1% SDS and its concentration was measured by NanoDrop 1000 Spectrophotometer.

### Plasmid constructs

Original 4.5SH RNA gene construct contained natural genomic 50 bp upstream sequence [30]. All 4.5SH gene-derived constructs also contained this upstream sequence. Mouse 4.5SI RNA gene Mmu1' construct [26] and its derivatives had 87 bp genomic upstream sequence. Nucleotide sequences of 4.5SH and 4.5SI RNA genes were changed by PCR. In the case of constructs *D*, *E*, *F*, and *I*, two-round PCR was used. The list of PCR primers is shown in Table S1. PCR products were cloned into pGEM-T (Promega). Plasmids were isolated using a variant of alkaline method [45], but without RNase treatment. All constructs were sequenced in order to avoid nucleotide substitutions introduced during PCR.

### Northern blot analysis

Equal amounts of cellular RNA were separated by denaturing electrophoresis in 6% PAAG and transferred onto Hybond-XL membrane (GE Healthcare UK Ltd., Buckinghamshire, England) by semidry electroblotting at 5 V for 1.5 h. The 4.5SH RNA, 4.5SI RNA and their chimeras were detected by hybridization with ^32^P-labeled probe obtained by PCR [15], [23] (see Fig. S1 for the primers). The blot was incubated overnight with the probe in 50% formamide, 5× Denhardt solution, 4× SSC, 1% SDS, and 0.1 mg/mL salmon sperm DNA at 42°C. The membrane was washed in 0.1× SSC and 0.1% SDS at 42°C, exposed against an X-ray film, and scanned by Cyclone phosphoroimager.

### Computer analysis

Secondary structure was predicted for small RNAs using the mFold 3.2 web server with default parameters [46]. The best prediction was used.

## Supporting Information

Figure S1 **Position of the PCR primers used in the preparation of templates.**
(DOC)

Table S1 **Primers used for PCR preparation of 4.5SH and 4.5SI-derived constructs.**
(DOC)
