# Supplementary material for: Complementarity of End Regions Increases the Lifetime of Small RNAs in Mammalian Cells
Source: PLoS One. 2012 Sep 12;7(9):e44157. doi: 10.1371/journal.pone.0044157 (PMC3440375; doi:10.1371/journal.pone.0044157)
Supplement: Figure S1 — Position of the PCR primers used in the preparation of templates. (DOC) [file pone.0044157.s001.doc]

**4.5SH RNA gene**

**1 10 20 30 40 50**

**GCCGGTAGTGGTGGCGCACGCCGGTAGGATTTGCTGAAGGAGGCAGAGGCAGG**

***SHdir***

**60 70 80 90**

**AGGATCACGAGTTCGAGGCCAGCCTGGGCTACACATTTTТТТ**

***SHrev***

**4.5SI RNA gene**

**1 10 20 30 40 50**

**GGGCTGGAGAGATGGCTCAGCCGTTAAAGGCTAGGCTCACAACCAAAAATATAA**

***SIdir***

**60 70 80 90**

**GAGTTCGGTTCCCAGCACCCACGGCTGTCTCTCCAGCCACCTTTT**

***SIrev***

**5S rRNA gene**

**1 10 20 30 40 50**

**GTCTACGGCCATACCACCCTGAACGCGCCCGATCTCGTCTGATCTCGGAAGCTA**

***5Sdir***

**60 70 80 90 100**

**AGCAGGGTCGGGCCTGGTTAGTACTTGGATGGGAGACCGCCTGGGAATACCGG**

**110 120**

**GTGCTGTAGGCTT**

***5Srev***

**Figure S1.**

**Position of the PCR primers (arrows) used in the preparation of templates (colored).** Labeling of the hybridization probes was performed by PCR using the templates, ^32^P-ATP, and the corresponding reverse primers (***SHrev, SIrev*** or ***5Srev***).
