# Supplementary material for: Complementarity of End Regions Increases the Lifetime of Small RNAs in Mammalian Cells
Source: PLoS One. 2012 Sep 12;7(9):e44157. doi: 10.1371/journal.pone.0044157 (PMC3440375; doi:10.1371/journal.pone.0044157)
Supplement: Table S1 — Primers used for PCR preparation of 4.5SH and 4.5SI-derived constructs. (DOC) [file pone.0044157.s002.doc]

| Construct | | Primer (name and nucleotide sequence) | Annealing temperature (°C) |
| --- | --- | --- | --- |
| А | | SH200dir TGTCGTCATCGTTGGTAG  SV2rev GAAAAAAATGTGTAGCC | 48 |
| В | | SH200dir  SH-d12rev AAAAAAGCTGGCCTCGAACT | 54 |
| С | | SH200dir  SH/SI-3’rev AAAAAAGGTGGCTGGAGAGCTGGCCTCGAA | 42 |
| D | Round I PCR1 | SH200dir  SH/SI-5’rev CATCTCTCCAGCCCAGCGAACGGGGCT | 48 |
|  | Round I PCR2 | SH/SI-5’dir GGGCTGGAGAGATGGCTCAGCCGGTAGGATTTG  SV2rev | 48 |
|  | Round II | SH200dir  SV2rev | 48 |
| E | Round I PCR 1 | SH200dir  SH/SIcorrev TTGTGAGCCTAGCCTTTAACGGCGTGCGCCAC | 46 |
|  | Round I PCR 2 | SH/SIcordir GCTCACAACCAAAAATATAAGAGTTCGAGGCC  SV2rev | 46 |
|  | Round II | SH200dir  SV2rev | 46 |
| F | Round I PCR 1 | SH200dir  SH/SI-3’rev | 48 |
|  | Round I PCR 2 | SH/SI-5’dir  SV2rev | 48 |
|  | Round II | SH200dir  SV2rev | 48 |
| G | | SH200dir  SH3’duprev AAAAAGCCGGTAGTGGTGGCGGCCTCGAACTC | 46 |
| H | | SI-87dir AGGAATTCGGGGAATGA  SIrev AAAAAAGGTGGCTGGAGAGACA | 50 |
| I | Round I  PCR 1 | SI-87dir  SI invertrev1 AGAGAGGTCGGCGCCCAGGAGGCAC | 50 |
|  | Round I  PCR 2 | SI invertdir GCCGACCTCTCTTGGCTCAGCCGTT  SIrev | 42 |
|  | Round II | SI-87dir  SIrev | 40 |
| J | | SI-87dir  S1 invertrev2 AAAAAAGGTCCGACCTCTCTCAGCCGTGGGTGC | 46 |
| K | Round I  PCR 1 | SI-87dir  SI invertrev1 | 50 |
|  | Round I  PCR 2 | SI invertdir  SI invertrev2 | 42 |
|  | Round II | SI-87dir  SI invertrev2 | 40 |

**Table S1.**

**Primers used for PCR preparation of 4.5SH and 4.5SI-derived constructs.**

4.5SH and 4.5SI-derived sequences are shown in red and blue respectively. The altered sequences are highlighted in green. Primers corresponding to 5’-flanking gene sequences are given in violet.
